# Site-Specific Variability in the Chemical Diversity of the Antarctic Red Alga *Plocamium cartilagineum*

**DOI:** 10.3390/md11062126

**Published:** 2013-06-14

**Authors:** Ryan M. Young, Jacqueline L. von Salm, Margaret O. Amsler, Juan Lopez-Bautista, Charles D. Amsler, James B. McClintock, Bill J. Baker

**Affiliations:** 1 Department of Chemistry and Center for Drug Discovery and Innovation, University of South Florida, Tampa, FL 33620, USA; E-Mails: ryanyoung1@mail.usf.edu (R.M.Y.); jsalm@mail.usf.edu (J.L.S.); 2 Department of Biology, University of Alabama at Birmingham, Birmingham, AL 35294, USA; E-Mails: mamsler@uab.edu (M.O.A.); amsler@uab.edu (C.D.A.); mcclinto@uab.edu (J.B.M.); 3 Department of Biological Sciences, University of Alabama, Tuscaloosa, AL 35487, USA; E-Mail: jlopez@ua.edu

**Keywords:** red algae, secondary metabolite variability, Antarctica, metabogenomic analysis

## Abstract

*Plocamium cartilagineum* is a common red alga on the benthos of Antarctica and can be a dominant understory species along the western Antarctic Peninsula. Algae from this region have been studied chemically, and like “*P. cartilagineum*” from other worldwide locations where it is common, it is rich in halogenated monoterpenes, some of which have been implicated as feeding deterrents toward sympatric algal predators. Secondary metabolites are highly variable in this alga, both qualitatively and quantitatively, leading us to probe individual plants to track the possible link of variability to genetic or other factors. Using *cox*1 and *rbc*L gene sequencing, we find that the Antarctic alga divides into two closely related phylogroups, but not species, each of which is further divided into one of five chemogroups. The chemogroups themselves, defined on the basis of Bray-Curtis similarity profiling of GC/QqQ chromatographic analyses, are largely site specific within a 10 km^2^ area. Thus, on the limited geographical range of this analysis, *P. cartilagineum* displays only modest genetic radiation, but its secondary metabolome was found to have experienced more extensive radiation. Such metabogenomic divergence demonstrated on the larger geographical scale of the Antarctic Peninsula, or perhaps even continent-wide, may contribute to the discovery of cryptic speciation.

## 1. Introduction

The rhodophyte *Plocamium cartilagineum* is widely reported in the world’s oceans, including the cold waters of the Arctic and Antarctic, temperate waters of Europe, Asia, Africa and the Americas, and tropical waters of the Pacific, Atlantic and Indian Oceans [1]. Systematists are of the opinion that multiple cryptic species are represented among these reports, and molecular techniques are being brought to bear on the problem [2]. Independent chemical analyses done in parallel with the distribution studies have identified well over 50 unique secondary metabolites [3]. More than 95% of metabolites reported from *P. cartilagineum* are polyhalogenated monoterpenes, and many of them bear pharmacological properties, such as cytotoxicity or antibiotic activity [4].

In Antarctica, *Plocamium cartilagineum* has a circumpolar distribution [5,6], although there is a great deal of genetic diversity within the taxon*.* The systematics and taxonomy of “*P. cartilagineum*” is in flux in Antarctica, as elsewhere [2]. Early workers [5,7,8,9] reported it as *P. coccineum*, which is now considered a synonym of *P. cartilagineum* [1]. The Subantarctic species *P. hookeri* and *P. secundatum* have also been reported from some locations along the western Antarctic Peninsula (WAP) [8]. Subsequently, it is believed that these morphological variants intergrade to the point that at present, all should be considered within *P. cartilagineum*, but their distinctiveness illustrates that there is notable morphological variability within Antarctic *P. cartilagineum* [10].

In recent field seasons at Palmer Station (64°46.5′S; 64°03.3′W), on the WAP, we have collected *P. cartilagineum* from shallow water habitats, where it can be locally dominant. Bulk collections of algae, comprising mixed populations from numerous collecting sites, produced extracts that were used in assays to ascertain levels of deterrence toward potential predators [11]. Field studies have identified halogenated monoterpenes with feeding deterrent effects toward the sympatric amphipod *Gondogeneia antarctica*, though lack of sufficient material hindered screening of the compounds against potential fish predators [12]. *P. cartilagineum* serves as host to several amphipod species, where they gain protection from fish predation, due to the physical structure of the algal thallus and feeding deterrent effects from metabolites found in its extracts [13]. Most of these amphipod species are similarly deterred by the extracts from consuming the alga [14], but in a mutualistic relationship, they graze endo- and epi-phytic algae from the surface of *P. cartilagineum*, inhibiting overgrowth that might otherwise limit the photosynthetic potential of the alga [15].

Collection-to-collection variability observed in the suite of secondary metabolites from WAP *P. cartilagineum* complicated tissue- and extract-based assays used in evaluating the extent to which these compounds protected the alga from predation, prompting us to profile individual plants. We report here a detailed metabogenomic analysis of site specificity and distribution of halogenated monoterpenes in two phylogroups of *P. cartilagineum* from the western Antarctic Peninsula.

## 2. Results and Discussion

**Figure 1:**
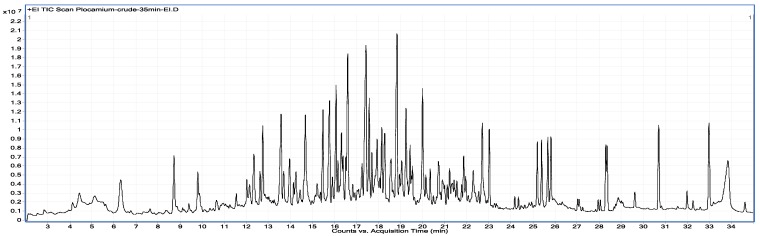
Electron impact (EIMS) total ion chromatogram (TIC) from a 35 min temperature gradient (60 °C to 300 °C) of a *Plocamium cartilagineum* lipophilic extract (3:1 dichloromethane/methanol). More than 50 resolved peaks are evident, most of which display halogen isotopic distribution patterns indicative of the polyhalogenated monoterpenes that *P. cartilagineum* is known to produce [3].

**Figure 2:**
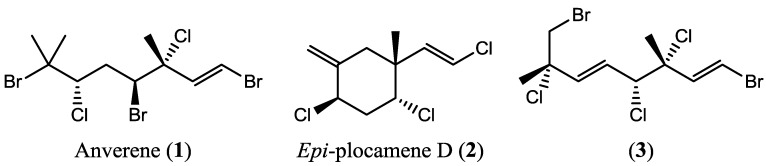
Representative western Antarctic Peninsula (WAP) *Plocamium cartilagineum* compounds [12,16], including the amphipod feeding deterrents [12] anverene (**1**) and *epi*-plocamene D (**2**). For comparison, anverene, under the same chromatographic conditions used for Figure 1, (**3**) elutes at about 19 min.

**Figure 3:**
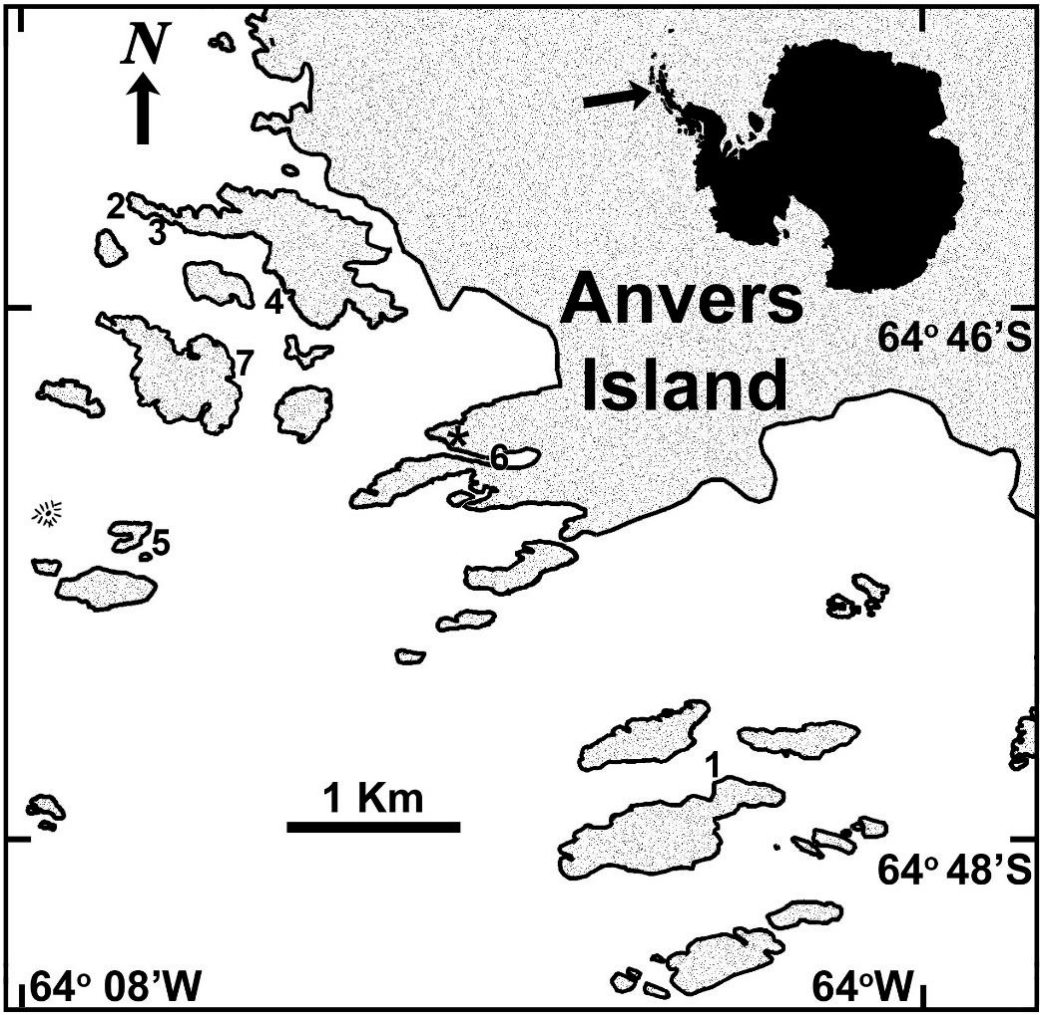
Collecting sites within the small boating range of Palmer Station, Antarctica (designated with an asterisk). (1) Hermit Island, algae collected at 19 m; (2) Norsel Point wall, 10 m; (3) Norsel Point cove, 6 m; (4) Old Palmer Station, 4 m; (5) Bahia Paraiso shipwreck, 17 m; (6) Hero Inlet, 6 m; (7) Litchfield Island, 12 m. The silhouette and arrow in the upper right indicate the location of Anvers Island along the Antarctic continent.

Collections of *Plocamium cartilagineum* that combine plant material collected from multiple sites within the small boating range around Palmer Station, Antarctica, display a breadth of secondary metabolites (e.g., Figure 1) rivaling even the 50 known halogenated monoterpenes in the published literature [3]. We have characterized a number of compounds (Figure 2) from these bulk collected samples over several years of field work at Palmer Station, though we noted the absence of some metabolites in some bulk collections and variability in others. To evaluate factors influencing such variability, we analyzed 21 individual algal samples collected in February 2012 from seven discrete collecting sites (Figure 3) within accessible dive sites near Palmer Station. We first analyzed the algal samples by GC/MS, which demonstrated distinct secondary metabolite profiles among the 21 individuals. To quantify and further understand the variability, we then conducted cluster analysis of the GC/MS data and genetic analysis of the algae using plastid *rbc*L and mitochondrial *cox*1 genes.

### 2.1. Chemical Analysis

**Figure 4:**
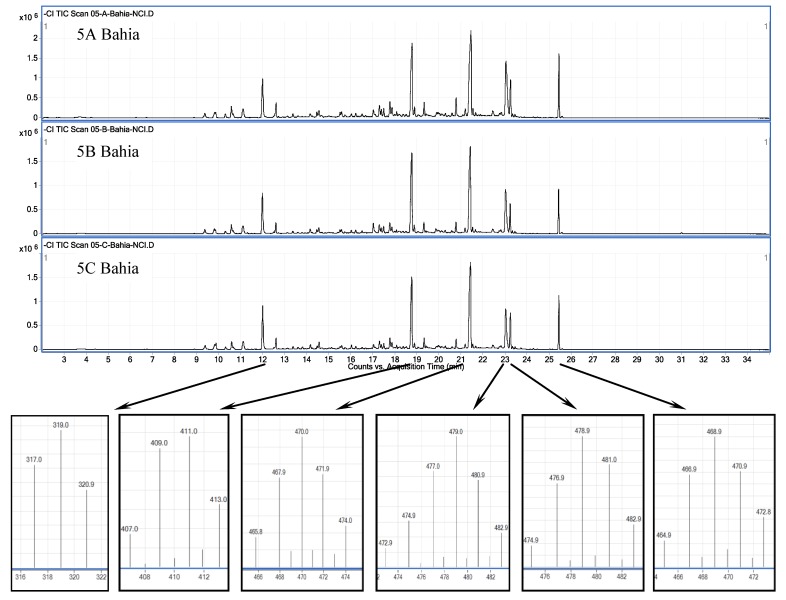
Negative chemical ionization (NCIMS) GC/MS chromatographic profile of three members of chemogroup 4 highlighting distinctive features (retention time, characteristic isotope distribution pattern of largest mass ion).

Analysis of individual plants by GC/QqQ negative chemical ionization (NCI) mass spectrometry, a technique especially well-suited to halogenated compounds [17], showed that chemodiversity varied significantly among some plants, but not others, leading us to assign secondary metabolite expression profiles as chemogroups. Figure 4, for example, illustrates chromatograms obtained from lipophilic (3:1 CH_2_Cl_2_/CH_3_OH) extracts of the three members of chemogroup 4, each individual of which (designated A, B and C in Figure 4) was collected near the wreck of the *Bahia Paraiso* (site 5, Figure 3). Six prominent features are visible in each chromatogram: (1) 12.0 min, 317/319/321 (~1:2:1) amu; (2) 18.6 min, 407/409/411/413 (~1:4:5:3) amu; (3) 21.4 min, 466/468/470/472/474 (~1:2:3:2:1) amu; (4) 23.0 min, 473/475/477/479/481/483 (~1:3:8:11:8:3) amu; (5) 23.3 min, 475/477/479/481/483 (~1:4:6:4:2) amu; and (6) 25.5 min, 465/467/469/471/473 (~2:6:9:7:3) amu. These masses and mass distributions, taken with the retention time and peak shape, are characteristic of the polyhalogenated monoterpene class of metabolites well known from this species [3]. Such characteristics distinguish these chromatographic features from the only known exception to *P. cartilagineum* monoterpene chemistry, which is a report of a series of four degraded sesquiterpene carboxylic acids [18].

**Figure 5:**
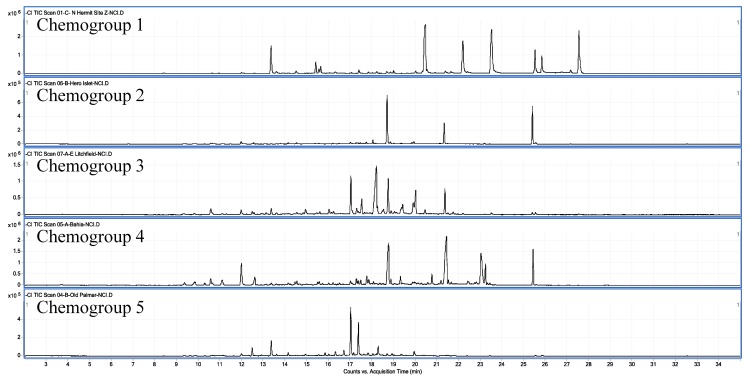
Negative chemical ionization (NCIMS) chromatographic profiles of secondary metabolome from five chemogroups of Palmer-area *Plocamium cartilagineum*.

Relative intensities of the chromatographic features observed in Figure 4, as well as minor features present in the chromatograms, track consistently among the three plants, identifying the individual plants as expressing the same secondary metabolome. Figure 5 compares one member from all five chemogroups. Notice that only one, chemogroup 2, shares more than one major feature with chemogroup 4’s major features (18.6, 21.4 and 25.4 min), although the relative intensities differ between shared features of chemogroup 2 and 4. In addition, two, chemogroups 1 and 5, share no major features with chemogroup 4. Figure 5 then displays the secondary metabolome that characterize chemogroups identified in this study. Further identification of metabolites is not possible at this time, due to a lack of standards for comparison. However, isotopic distribution patterns like those described for chemogroup 4 clearly identify the metabolites in this study as polyhalogenated secondary metabolites.

### 2.2. Genetic Analysis

**Figure 6:**
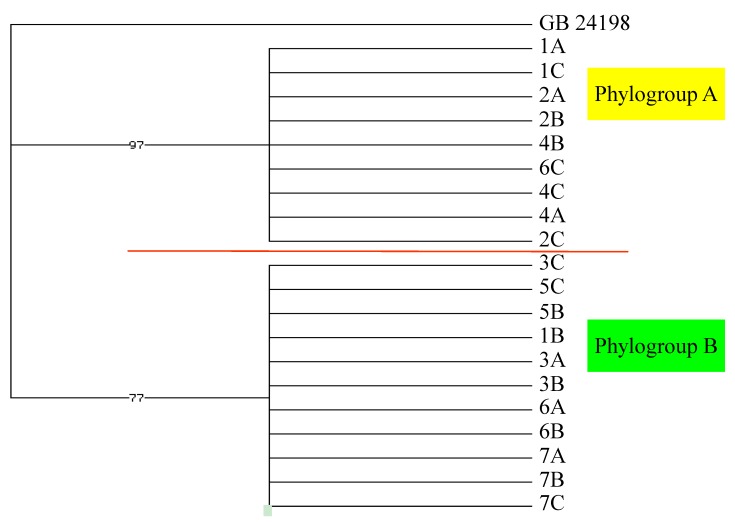
*cox*1 mitochondrial lineage of Palmer-area *Plocamium cartilagineum* individuals identified by site and collection sequence (1A…7C). Included for comparison is a King George Island, Antarctica, sequence (GB 24198) from GenBank.

**Figure 7:**
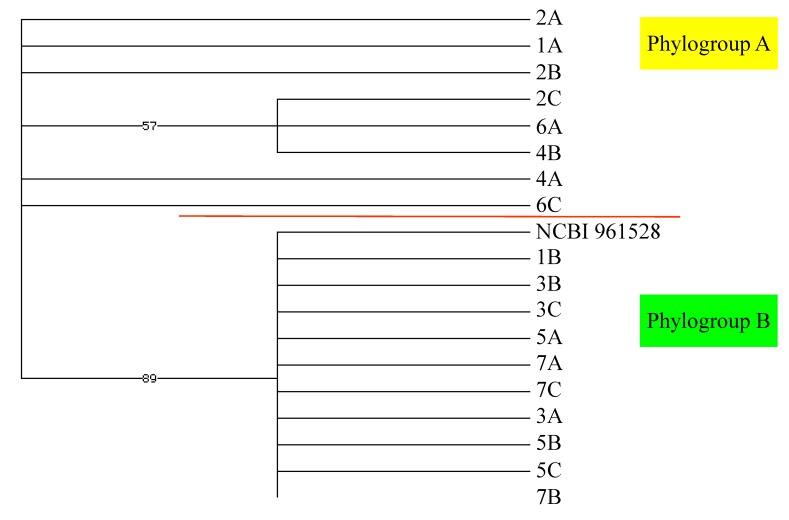
*rbc*L lineage of Palmer-area *Plocamium cartilagineum* individuals identified by site and collection sequence (1A…7C). Included for comparison is a King George Island, Antarctica, sequence (NCBI 951528) from GenBank.

Although there was morphological variation within our 21 individual algal specimens, there were no clear morphological groupings. However, based on the known cryptic diversity within *P. cartilagineum*, we chose to sequence the *rbc*L and *cox*1 genes to examine genetic diversity within our collection [2]. The *cox*1 genes from our algal samples diverged into two clades (Figure 6), designated phylogroups A and B, with nine and eleven members, respectively. The *rbc*L analysis (Figure 7) found the same members in phylogroup B, with the exception of one alga, designated Hero Inlet sample A (6A), which clustered with phylogroup A. The *rbc*L gene of phylogroup A was distinct from that of phylogroup B, although five of the eight members claded independently (Figure 7). The divergence between genotypes is 2.1% or less for *cox*1 (Figure S1) and 1.1% or less for *rbc*L (Figure S2). Based on these small sequence differences, the specimens represented in the dataset strongly reflect conspecificity. These values are in agreement with previous reports on percent differences among individuals of the same species for red algae using *cox*1 and *rbc*L [19,20,21]. However, the observed divergence could represent an early stage of cryptic speciation.

### 2.3. Metabogenetic Characterization of *Plocamium cartilagineum*

**Figure 8:**
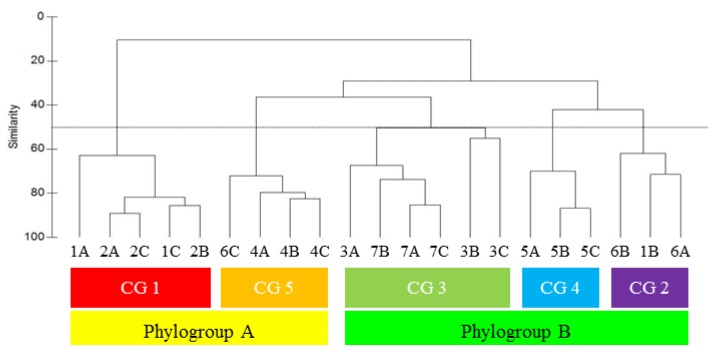
Bray-Curtis similarity dendrogram showing the relationship of the metabolomic profiles between algal collection sites (labeled by site number (1–7) and sample replicate (A–C)). Algae clustering with greater than 50% similarity (dotted line) were assigned as chemogroups 1–5 (CG 1–5). Chemogroups further separated into the two phylogroups A and B.

To quantify the chemogroups that were evident from visual inspection (see 2.1. Chemical Analysis, above), GC/MS data was analyzed statistically. The same five chemogroups distinguish themselves from one another at approximately 50% Bray-Curtis similarity (Figure 8). Chemogroup 1 (red bar) differs most from other chemogroups, displaying approximately 10% Bray-Curtis similarity to both its sister chemogroup in phylogroup A and to phylogroup B. Chemogroup 5 (orange bar) is equally distant from its sister chemogroup in phylogroup A, chemogroup 1, as it is to phylogroup B, with approximately 30% similarity to both clusters. Phylogroup B, on the other hand, forms a distinct clade of three chemogroups, approximately 40% similar to one another. Chemogroups 2 (purple bar) and 4 (blue bar) diverge at the 40% similarity delineation, but separate from chemogroup 3 (green bar) at 30% similarity. Increasing Bray-Curtis similarity above 50% results in further diversification of chemogroups that is unsupportable by other measures of similarity. Such specificity of metabolites to phylogroups defines their metabogenomic relatedness.

### 2.4. Site Specificity among *Plocamium cartilagineum* Metabogenetic Groups

Algal collection sites were within 3 km of Palmer Station, an area encompassing approximately 10 km^2^ (see Figure 3 for numbered sites). Within a collecting site, three individual algal specimens, labeled A–C, were collected at the same depth and within 5 m (most within 2 m) of one another laterally. With the exception of one Hermit Island alga (1B) and one Hero Inlet alga (6C), algal collection sites were phylo- and chemo-group specific. Individual plants from Old Palmer (4A–C), Bahia (5A–C) and Litchfield (7A–C) were tightly clustered within a chemogroup (CG 5, 4 and 3, respectively), with Bray-Curtis similarity >70% at each site. Individual algae from Norsel Point wall (2A–C) and Norsel Point Cove (3A–C), which lie approximately 125 m from one another, interestingly clustered with their site cohort, but the two cohorts differed in both phylogroup (A *vs.* B, respectively) and chemogroup (1 *vs.* 3, respectively). 

Such site specificity is surprising. Similar studies of the red alga *Portieria hornemannii*, encompassing only a slightly larger geographical range, found no reproducible site specificity among secondary metabolites [22,23]. However, another study of *P. hornemannii* found that light availability, which could potentially vary from site-to-site, might influence the quantitation of a halogenated monoterpene [24]. The effects of varying light and temperature on the production of three (of many) halogenated monoterpenes produced by Chilean *P. cartilagineum* have been studied [25], and it was reported that temperature tended to have the greater effect. However, this was only statistically significant for one compound, whose production also varied with light level at one temperature. Light (PAR; photosynthetically active radiation) is generally considered the most important environmental variable for Antarctic macroalgae, particularly since inorganic nutrient levels are rarely, if ever, growth limiting and annual temperature variations are small [7,26]. PAR, along with ultraviolet radiation and wave surge, vary with depth. Our collections were made at a single depth per site, with depths ranging from 4 to 19 m (Figure 3), so it is possible that some of the site to site variation could actually reflect an influence of depth. Within phylogroup A, chemogroup 1 individuals were collected at either 10 or 19 m, while chemogroup 5 individuals came from 4 or 6 m. However, no such pattern is apparent in phylogroup B, and even with phylogroup A, the limited sample size and obvious additional correlation with the collection site precludes definitive conclusions.

Another factor that may vary across both depth and site is predation pressure. By far the most abundant grazers in the community are amphipods [27,28,29]. As part of a current project, we have collected amphipods associated with *P. cartilagineum* at multiple sites and depths in this study area. Although counts of individuals have not been made, the fauna is qualitatively similar across all collections (same general group of species). However, there do appear to be quantitative differences across sites in the numbers of at least some specific amphipod species (M. Amsler personal observation). The possibility that these differences are either causes or consequences of the chemical diversity remains to be explored.

Light and wave energy also vary with exposure, with more exposed sites generally having clearer, open ocean water more frequently. Greater distance from glaciers (and their silty melt water) also generally corresponds with clearer waters. However, no consistent patterns emerge when considering these factors, for example, algae from the most exposed site, Norsel Point wall (site 2) group with two individuals from a moderately protected site at Hermit Island (site 1). The Hermit site has less glacial input, so clearer waters than some of the other protected sites, but not the other Hermit individual (1B) groups with two individuals from Hero Inlet, which is by far the most protected site with the most glacial influence and most turbid water. The other Hero Inlet individual (6C), groups with individuals from Old Palmer, which is probably the second most protected and second most glacial-influenced site, although in both cases, much less so than Hero Inlet.

The only chemogroup that was restricted to a single site was chemogroup 5 at the *Bahia Paraiso* shipwreck (Figure 3). This site should be considered disturbed. The ship sank in 1989 and, while eventually de-fueled, a small amount of fuel continues to leak from the ship, imparting water-borne pollutants. The algae were collected less than 10 m from the ship hull, so trace inputs of metals or materials from paint, *etc.*, could also be present. However, the macroalgal and associated grazer community is not qualitatively different at that collection site from many other sites in the study area. Outside of the possibility that the trace pollutants could impact some specific grazer species more than others, it is not obvious why the trace pollutants themselves would induce production of a specific chemogroup or select for the survival of individuals constitutively producing a particular chemogroup.

We are aware of two reports of secondary metabolite variations in red algae that are related to life history stage [22,30], including one [22] where different chemogroups correlated with different life history stages. Only a minority of *P. cartilagineum* from our study area are routinely collected with reproductive structures, and none of the individual *P. cartilagineum* in our survey were fertile, so the potential relationship between their life history stage and chemogroup could not be determined. In both cases (sites 1 and 6) where a single site had multiple chemogroups, the algae were also in different phylogroups. However, it is possible that some of the within-chemogroup diversity in secondary metabolites could be related to algal life history stage.

## 3. Experimental Section

### 3.1. Biological Material

A 24.7 kg sample of *P. cartilagineum* was hand collected by scuba diving between February and April, 2010, from multiple dive sites around Palmer Station on Anvers Island, Antarctica (64°46.5′S; 64°03.3′W). Periodic collections were cleaned of foreign materials in the Palmer Station aquarium, then frozen at −20 °C, with subsequent collections combined to produce this mixed population that was used for chemical isolation and characterization studies at our home institutions. Individual thalli used in this study were collected in late February 2012 from seven dive sites in the vicinity of Palmer Station (Figure 3), with three individual plants sampled at each site and all twenty-one samples separately cleaned, photographed, frozen and then shipped to our home institutions for analysis. Individuals at each site were collected at the same depth (±1 m; usually ±0.3 m) and no more than 5 m laterally (usually <2 m laterally).

### 3.2. Chemical Analysis

#### 3.2.1. Algal Extraction and Monoterpene Purification

Mixed population algae from the 2010 collection were thawed, then extracted in 3:1 dichloromethane/methanol. The concentrated lipophilic extract was examined by GC/MSm as described below, then purified by repeated medium pressure silica gel chromatography (100% hexane to 100% ethyl acetate on 4 g or 12 g Teledyne/Isco silica gel cartridges)m followed by repeated high pressure liquid chromatography (10 × 250 mm Sunfire column, 5 mL/min 100% pentane). Anverene (**1**), *epi*-plocamene D (**2**), and (1*E*,3*R*,4*R*,5*E*,7*S*)-1,8-dibromo-3,4,7-trichloro-3,7-dimethylocta-1,5-diene (**3**) were characterized by comparison of their ^1^H and ^13^C NMR spectra to that reported in the literature [12,16]. Compounds **1** and **3** were also subject to X-ray crystallographic analysis.

The 21 algal specimens from the 2012 collection were individually extracted, as described above, and used directly for GC/MS analysis.

#### 3.2.2. GC/MS Analysis

Each algal extract was filtered (0.45 μm PTFE membrane), concentrated under a stream of nitrogen gas, then transferred to a GC/MS sample vial in methanol to a final concentration of 1 mg/mL. Extracts were analyzed on an Agilent 7980A GC interfaced to an Agilent 7000 series QqQ mass spectrometer operating in either electron ionization (EI) or chemical ionization (CI) mode, the latter using methane at 2 mL/min as the ionization gas. Injections of 1 μL of the algal extract solution were vaporized on the preheated splitless inlet at 250 °C, then introduced onto an HP-5ms column (30 m × 0.25 mm i.d.) using a 35 min temperature gradient (initial oven temperature of 100 °C, held for 2 min, heated to a final temperature of 250 °C at a rate of 5 °C/min, then held at final temperature for a further three minutes). Helium was used as a carrier gas at a constant flow rate of 1 mL/min.

### 3.3. Phylogenetic Analysis

DNA was extracted from silica dried tissue (~10 mg) using the Qiagen DNeasy Plant mini kit. Specific primers amplified the *cox*1 mitochondrial gene [31] and the plastid bound *rbc*L gene [32]. PCR amplification was conducted in an Eppendorf Mastercycler. *cox*1 amplification began with an initial denaturation at 95 °C for 2 min, followed by 5 cycles of 30 s at 95 °C, 30 s at 45 °C and 1 min at 72 °C; then 35 cycles of 30 s at 95 °C, 30 s at 46.5 °C and 1 min at 72 °C; and finally, a 7 min extension at 72 °C. *rbc*L amplification initiated with a 3 min denaturation at 94 °C, then 40 cycles of 90 s at 94 °C, 2 min at 37 °C, 3 min at 72 °C, terminating with 4 min extension at 72 °C. Duplicate PCR products were pooled and purified with Promega Wizard prior to sequencing on an ABI PRISM 3730. Six-hundred sixty base pairs were read for *cox*1 and 890 bp for *rbc*L. Sequences were aligned by eye and with the software Geneious (version 5.1.7, created by Biomatters) using the MUSCLE plugin [33]. Alignments were exported in NEXUS format and analyzed separately using jModelTest2 [34,35]. The GTR + I + gamma model was selected for both *cox*1 and *rbc*L. Maximum likelihood phylogenetic analysis was performed on the CIPRES Science Gateway [36] using GARLI 2.0 [37] with 100 bootstrap replicates. A neighbor-joining tree for each marker was constructed in Geneious using default settings, and the resulting pairwise distance matrix was exported as an image file. GenBank submission numbers for *cox*1 A and B and *rbc*L A and B are KF158990, KF158991, KF158993 and KF158992 respectively.

### 3.4. Metabolomic Data Processing and Multivariate Analysis

The GC/MS data was analyzed in Agilent’s MassHunter Qualitative B5.01, where 43 recurring compounds were identified between 10 and 30 min throughout the 21 samples. The height of these peaks relative (>10%) to that of the most abundant peak within each chromatogram was calculated and input into PRIMER-6 for statistical analysis. The peak height data for all peaks >10% of the largest peak were analyzed for resemblance using Bray-Curtis similarity and clustered using the group average.

## 4. Conclusions

*Plocamium* *cartilagineum* collected from the vicinity of Palmer Station, Antarctica, diverges both chemically and genetically, but should still be considered as a single species. We see site-specific differences in both phylogeny and secondary metabolism between closely spaced sites (e.g., 125 m, Norsel Point wall *vs.* Norsel Point cove) and also similarities between more distantly related sites (e.g., 4 km, Norsel Point wall *vs.* Hermit Island). Observable environmental factors do not appear to influence site specificity. *P. cartilagineum* on the WAP may be undergoing cryptic speciation as is seen elsewhere, and its chemical diversity could be either a partial consequence or even a partial driver of that, but a broader view that incorporates similar data from algae more distant on the WAP, or from other sites on the continent, needs to be considered.

## Supplementary Files

Supplementary File 1 Supplementary Information (PDF, 50 KB)
