# Supplementary material for: Site-Specific Variability in the Chemical Diversity of the Antarctic Red Alga Plocamium cartilagineum
Source: Mar Drugs. 2013 Jun 14;11(6):2126–39. doi: 10.3390/md11062126 (PMC3721224; doi:10.3390/md11062126)
Supplement: Supplementary File 1 — Supplementary Information (PDF, 50 KB) [file marinedrugs-11-02126-s001.pdf]

Supplementary Information

Figure S1. Percentage divergence among *P. cartilagineum* individuals based on *cox*1 gene.

	SANT-Algae-...	1aCOX	1Ccox	2Acox	2Bcox	4Bcox	6CCox	4Ccox	4Acox	2CcoxR-coxR	3CcoxR-coxR	5Ccox	5Bcox	1Bcox	3AcoxR-coxR	3Bcox	6ACOX	6Bcox	7Acox	7Bcox	7Ccox	FlatPlo
SANT-Algae-24198JF271583.1		0.069	0.069	0.069	0.069	0.069	0.069	0.069	0.069	0.069	0.069	0.071	0.069	0.069	0.069	0.069	0.069	0.069	0.069	0.069	0.069	0.069
1aCOX	0.069		0.000	0.000	0.000	0.000	0.000	0.000	0.000	0.000	0.021	0.021	0.019	0.019	0.019	0.019	0.019	0.019	0.019	0.019	0.019	0.019
1Ccox	0.069	0.000		0.000	0.000	0.000	0.000	0.000	0.000	0.000	0.021	0.021	0.019	0.019	0.019	0.019	0.019	0.019	0.019	0.019	0.019	0.019
2Acox	0.069	0.000	0.000		0.000	0.000	0.000	0.000	0.000	0.000	0.021	0.021	0.019	0.019	0.019	0.019	0.019	0.019	0.019	0.019	0.019	0.019
2Bcox	0.069	0.000	0.000	0.000		0.000	0.000	0.000	0.000	0.000	0.021	0.021	0.019	0.019	0.019	0.019	0.019	0.019	0.019	0.019	0.019	0.019
4Bcox	0.069	0.000	0.000	0.000	0.000		0.000	0.000	0.000	0.000	0.021	0.021	0.019	0.019	0.019	0.019	0.019	0.019	0.019	0.019	0.019	0.019
6CCox	0.069	0.000	0.000	0.000	0.000	0.000		0.000	0.000	0.000	0.021	0.021	0.019	0.019	0.019	0.019	0.019	0.019	0.019	0.019	0.019	0.019
4Ccox	0.069	0.000	0.000	0.000	0.000	0.000	0.000		0.000	0.000	0.021	0.021	0.019	0.019	0.019	0.019	0.019	0.019	0.019	0.019	0.019	0.019
4Acox	0.069	0.000	0.000	0.000	0.000	0.000	0.000	0.000		0.000	0.021	0.021	0.019	0.019	0.019	0.019	0.019	0.019	0.019	0.019	0.019	0.019
2CcoxR-coxR	0.069	0.000	0.000	0.000	0.000	0.000	0.000	0.000	0.000		0.021	0.021	0.019	0.019	0.019	0.019	0.019	0.019	0.019	0.019	0.019	0.019
3CcoxR-coxR	0.069	0.021	0.021	0.021	0.021	0.021	0.021	0.021	0.021	0.021		0.004	0.002	0.002	0.002	0.002	0.002	0.002	0.002	0.002	0.002	0.002
5Ccox	0.071	0.021	0.021	0.021	0.021	0.021	0.021	0.021	0.021	0.021	0.004		0.002	0.002	0.002	0.002	0.002	0.002	0.002	0.002	0.002	0.002
5Bcox	0.069	0.019	0.019	0.019	0.019	0.019	0.019	0.019	0.019	0.019	0.002	0.002		0.000	0.000	0.000	0.000	0.000	0.000	0.000	0.000	0.000
1Bcox	0.069	0.019	0.019	0.019	0.019	0.019	0.019	0.019	0.019	0.019	0.002	0.002	0.000		0.000	0.000	0.000	0.000	0.000	0.000	0.000	0.000
3AcoxR-coxR	0.069	0.019	0.019	0.019	0.019	0.019	0.019	0.019	0.019	0.019	0.002	0.002	0.000	0.000		0.000	0.000	0.000	0.000	0.000	0.000	0.000
3Bcox	0.069	0.019	0.019	0.019	0.019	0.019	0.019	0.019	0.019	0.019	0.002	0.002	0.000	0.000	0.000		0.000	0.000	0.000	0.000	0.000	0.000
6ACOX	0.069	0.019	0.019	0.019	0.019	0.019	0.019	0.019	0.019	0.019	0.002	0.002	0.000	0.000	0.000	0.000		0.000	0.000	0.000	0.000	0.000
6Bcox	0.069	0.019	0.019	0.019	0.019	0.019	0.019	0.019	0.019	0.019	0.002	0.002	0.000	0.000	0.000	0.000	0.000		0.000	0.000	0.000	0.000
7Acox	0.069	0.019	0.019	0.019	0.019	0.019	0.019	0.019	0.019	0.019	0.002	0.002	0.000	0.000	0.000	0.000	0.000	0.000		0.000	0.000	0.000
7Bcox	0.069	0.019	0.019	0.019	0.019	0.019	0.019	0.019	0.019	0.019	0.002	0.002	0.000	0.000	0.000	0.000	0.000	0.000	0.000		0.000	0.000
7Ccox	0.069	0.019	0.019	0.019	0.019	0.019	0.019	0.019	0.019	0.019	0.002	0.002	0.000	0.000	0.000	0.000	0.000	0.000	0.000	0.000		0.000
FlatPlo	0.069	0.019	0.019	0.019	0.019	0.019	0.019	0.019	0.019	0.019	0.002	0.002	0.000	0.000	0.000	0.000	0.000	0.000	0.000	0.000	0.000	

Figure S2. Percentage divergence among *P. cartilagineum* individuals based on *rbc*L gene.

	2ArbcL	1ArbcL	2BrbcL	2CrbcL	4ArbcL	6ArbcL	6C2rbcL	4BrbcL	ncbi961528(K...	1BrbcL	3BrbcL	3CrbcL	5ArbcL	7ArbcL	FlatrbcL	7CPlorbcL	3ArbcL	5BrbcL	5CrbcL	7BrbcL
2ArbcL		0.003	0.003	0.005	0.003	0.003	0.003	0.004	0.011	0.007	0.008	0.007	0.008	0.007	0.007	0.007	0.007	0.007	0.007	0.008
1ArbcL	0.003		0.000	0.001	0.000	0.000	0.000	0.000	0.008	0.003	0.005	0.003	0.005	0.003	0.003	0.003	0.003	0.003	0.003	0.003
2BrbcL	0.003	0.000		0.001	0.000	0.000	0.000	0.000	0.008	0.003	0.005	0.003	0.005	0.003	0.003	0.003	0.003	0.003	0.003	0.003
2CrbcL	0.005	0.001	0.001		0.001	0.000	0.001	0.000	0.009	0.005	0.006	0.005	0.006	0.005	0.005	0.005	0.005	0.005	0.005	0.003
4ArbcL	0.003	0.000	0.000	0.001		0.000	0.000	0.000	0.008	0.003	0.005	0.003	0.005	0.003	0.003	0.003	0.003	0.003	0.003	0.003
6ArbcL	0.003	0.000	0.000	0.000	0.000		0.000	0.000	0.008	0.003	0.005	0.003	0.005	0.003	0.003	0.003	0.003	0.003	0.003	0.003
6C2rbcL	0.003	0.000	0.000	0.001	0.000	0.000		0.000	0.008	0.003	0.005	0.003	0.005	0.003	0.003	0.003	0.003	0.003	0.003	0.003
4BrbcL	0.004	0.000	0.000	0.000	0.000	0.000	0.000		0.005	0.003	0.003	0.003	0.003	0.003	0.003	0.003	0.003	0.003	0.003	0.003
ncbi961528(KGI)	0.011	0.008	0.008	0.009	0.008	0.008	0.008	0.005		0.005	0.006	0.005	0.006	0.005	0.005	0.005	0.005	0.005	0.005	0.002
1BrbcL	0.007	0.003	0.003	0.005	0.003	0.003	0.003	0.003	0.005		0.001	0.000	0.001	0.000	0.000	0.000	0.000	0.000	0.000	0.000
3BrbcL	0.008	0.005	0.005	0.006	0.005	0.005	0.005	0.003	0.006	0.001		0.001	0.002	0.001	0.001	0.001	0.001	0.001	0.001	0.000
3CrbcL	0.007	0.003	0.003	0.005	0.003	0.003	0.003	0.003	0.005	0.000	0.001		0.001	0.000	0.000	0.000	0.000	0.000	0.000	0.000
5ArbcL	0.008	0.005	0.005	0.006	0.005	0.005	0.005	0.003	0.006	0.001	0.002	0.001		0.001	0.001	0.001	0.001	0.001	0.001	0.000
7ArbcL	0.007	0.003	0.003	0.005	0.003	0.003	0.003	0.003	0.005	0.000	0.001	0.000	0.001		0.000	0.000	0.000	0.000	0.000	0.000
FlatrbcL	0.007	0.003	0.003	0.005	0.003	0.003	0.003	0.003	0.005	0.000	0.001	0.000	0.001	0.000		0.000	0.000	0.000	0.000	0.000
7CPlorbcL	0.007	0.003	0.003	0.005	0.003	0.003	0.003	0.003	0.005	0.000	0.001	0.000	0.001	0.000	0.000		0.000	0.000	0.000	0.000
3ArbcL	0.007	0.003	0.003	0.005	0.003	0.003	0.003	0.003	0.005	0.000	0.001	0.000	0.001	0.000	0.000	0.000		0.000	0.000	0.000
5BrbcL	0.007	0.003	0.003	0.005	0.003	0.003	0.003	0.003	0.005	0.000	0.001	0.000	0.001	0.000	0.000	0.000	0.000		0.000	0.000
5CrbcL	0.007	0.003	0.003	0.005	0.003	0.003	0.003	0.003	0.005	0.000	0.001	0.000	0.001	0.000	0.000	0.000	0.000	0.000		0.000
7BrbcL	0.008	0.003	0.003	0.003	0.003	0.003	0.003	0.003	0.002	0.000	0.000	0.000	0.000	0.000	0.000	0.000	0.000	0.000	0.000	
